# Wood density is related to aboveground biomass and productivity along a successional gradient in upper Andean tropical forests

**DOI:** 10.3389/fpls.2023.1276424

**Published:** 2023-11-09

**Authors:** Dennis Castillo-Figueroa, Andrés González-Melo, Juan M. Posada

**Affiliations:** Biology Department, Faculty of Natural Sciences, Universidad del Rosario, Bogota, Colombia

**Keywords:** montane forest, carbon cycle, Colombian Andes, functional traits, community weighted mean

## Abstract

Wood density (WD) is a key functional trait related to ecological strategies and ecosystem carbon dynamics. Despite its importance, there is a considerable lack of information on WD in tropical Andean forests, particularly regarding its relationship with forest succession and ecosystem carbon cycling. Here, we quantified WD in 86 upper Andean tree and shrub species in central Colombia, with the aim of determining how WD changes with forest succession and how it is related to productivity. We hypothesized that WD will increase with succession because early successional forests will be colonized by acquisitive species, which typically have low WD, while the shaded understory of older forests should favor higher WD. We measured WD in 481 individuals from 27 shrub and 59 tree species, and quantified aboveground biomass (AGB), canopy height, net primary production (NPP) and species composition and abundance in 14, 400-m^2^, permanent plots. Mean WD was 0.513 ± 0.114 (g/cm^3^), with a range between 0.068 and 0.718 (g/cm^3^). Shrubs had, on average, higher WD (0.552 ± 0.095 g/cm^3^) than trees (0.488 ± 0.104 g/cm^3^). Community weighted mean WD (CWMwd) decreased with succession (measured as mean canopy height, AGB, and basal area); CWMwd also decreased with aboveground NPP and stem growth. In contrast, the percentage of NPP attributed to litter and the percent of shrubs in plots increased with CWMwd. Thus, our hypothesis was not supported because early successional forests had higher CWMwd than late successional forests. This was related to a high proportion of shrubs (with high WD) early in succession, which could be a consequence of: 1) a low seed availability of trees due to intense land use in the landscape and/or 2) harsh abiotic conditions early in succession that filter out trees. Forest with high CWMwd had a high %NPP attributed to litter because they were dominated by shrubs, which gain little biomass in their trunks. Our findings highlight the links between WD, succession and carbon cycling (biomass and productivity) in this biodiversity hotspot. Thus, WD is an important trait that can be used to understand upper Andean forest recovery and improve forest restoration and management practices.

## Introduction

Wood density (WD) is a key functional trait for understanding tree functioning and community ecology (Chave et al., 2005; Chave et al., 2009; Oliveira et al., 2019; González-Melo, 2021). It is also important in ecosystem studies (Padilha and Marco Júnior, 2018), that require estimates of aboveground biomass, productivity, and carbon emissions (Fearnside, 1997; Muller-Landau, 2004; Chave et al., 2009). In an eco-physiological context, high WD has been related to lower water transport capacity in trunks and branches (Stratton et al., 2000; Scholz et al., 2007; Meinzer et al., 2008), but higher drought tolerance (Markesteijn et al., 2011). WD has also an inverse relationship with growth (Muller-Landau, 2004; Osunkoya et al., 2007; Kraft et al., 2010), mortality rates (Chave et al., 2009), and recruitment rates (Suzuki, 1999; Wright et al., 2003; King et al., 2006). Likewise, some studies have indicated that WD plays an important role in ecosystem processes as it is related to aboveground biomass (AGB), the rate of biomass accumulation (Baker et al., 2004; Chave et al., 2005; Stegen et al., 2009; Oliveira et al., 2019) and wood decomposition (Aryal et al., 2022). AGB is also related to forest successional status (i.e., age; Brown and Lugo, 1990; Garnier et al., 2004; Garnier et al., 2016; Chazdon et al., 2016; Poorter et al., 2021a) and successional changes in WD vary between ecosystems (Poorter et al., 2021b). For instance, WD increases with forest age in lowland tropical rain forests due to a shade tolerance filter later in succession (Poorter et al., 2021b). Another aspect to consider when studying WD is life form. In particular, WD can differ considerably between shrubs and trees (Martínez-Cabrera et al., 2009) because these functional groups have different height, and can vary in their resource needs (i.e., water, light or nutrients; Martínez-Cabrera et al., 2009).

Several studies related to WD have made significant contributions to both regional (Chave et al., 2006; Oliveira et al., 2019; Báez et al., 2022) and global trait databases (Chave et al., 2009; Zanne et al., 2009; Henry et al., 2013; Kattge et al., 2020). In the tropics, the bulk of research on WD has been carried out in lowland ecosystems like the Amazonian rainforest (Nogueira et al., 2008), the Brazilian Cerrado (Roitman et al., 2018), Panamanian lowland forest (Muller-Landau, 2004; Wright et al., 2010) and the Atlantic forest (Padilha and Marco Júnior, 2018), among others. Nevertheless, there is a considerable gap in our knowledge of WD variations in tropical montane forests, particularly in geographical regions above 2500 meters elevation (Álvarez-Dávila et al., 2017).

Tropical mountain forests play pivotal roles in biodiversity conservation, and water and carbon cycles (Galbraith et al., 2014; Duque et al., 2021). In particular, the upper Andes Mountains of Northern South America (2600-3200m) represent a global hotspot of biological diversity due to their significant of endemism and significant of transformation (Hernández-Camacho et al., 1992; Myers et al., 2000; Myster, 2021). However, Upper Andean Tropical Forests (UATF; above ca. 2500 m elevation) are among the least known ecosystems (Etter and van Wyngaarden, 2000), especially in relation to species functional traits, ecological succession, and ecosystem processes (Castillo-Figueroa, 2021). These forests have experienced extensive habitat loss due to large-scale deforestation and conversion of lands to agriculture and urban areas (Etter and van Wyngaarden, 2000; Etter and Villa, 2000; Etter et al., 2008; Etter et al., 2021), that has resulted in a mosaic of forests with different successional status, ranging from early-secondary to small and isolated old-growth forest patches (Rubiano et al., 2017; Calbi et al., 2020; Calbi et al., 2021; Hurtado-M et al., 2021).

Trait-based ecology predicts that WD should increase during succession, because in the first successional stages forests are commonly colonized by species with a high resource acquisition strategy, with rapid growth rates but low WD, which allow them to take advantage of high light and nutrient availability (Chave et al., 2009; Chazdon, 2014; Garnier et al., 2016; but see Poorter et al., 2021b). In contrast, old-growth forests are usually colonized by species with conservative strategies, with low growth rate and high WD that allow them to tolerate understory conditions (Chao et al., 2008; Chave et al., 2009; Poorter et al., 2021b). Yet, evidence supporting this prediction comes mainly from tropical lowland rainforests, and it is still unclear whether this pattern holds true for UATF. In this sense, the relationship of WD with forest successional status (measured by structural attributes such as AGB, basal area, and canopy height) would represent a step forward in our understanding of UATF function and regeneration.

In this study, we measured WD for 86 species along a successional gradient in UATF. We compared WD between life forms and assessed how forest structural attributes and net primary production (NPP) are related to community-weighted mean wood density (CWMwd) along a successional gradient. We addressed the following questions: (1) What is the range of variation of WD in species from UATF in comparison to regional and global databases? (2) Does WD differ between shrubs and trees? (3) How is CWMwd related to forest successional status (using basal area, AGB, and canopy height as proxies of succession) and to NPP along a successional gradient? Given that the studied UATF are exposed to mesic conditions, our main hypothesis was that CWMwd will increase with successional status because high resource availability early in succession will favor acquisitive species, that will be gradually replaced by species with more conservative traits.

## Materials and methods

### Study area

**Figure 1:**
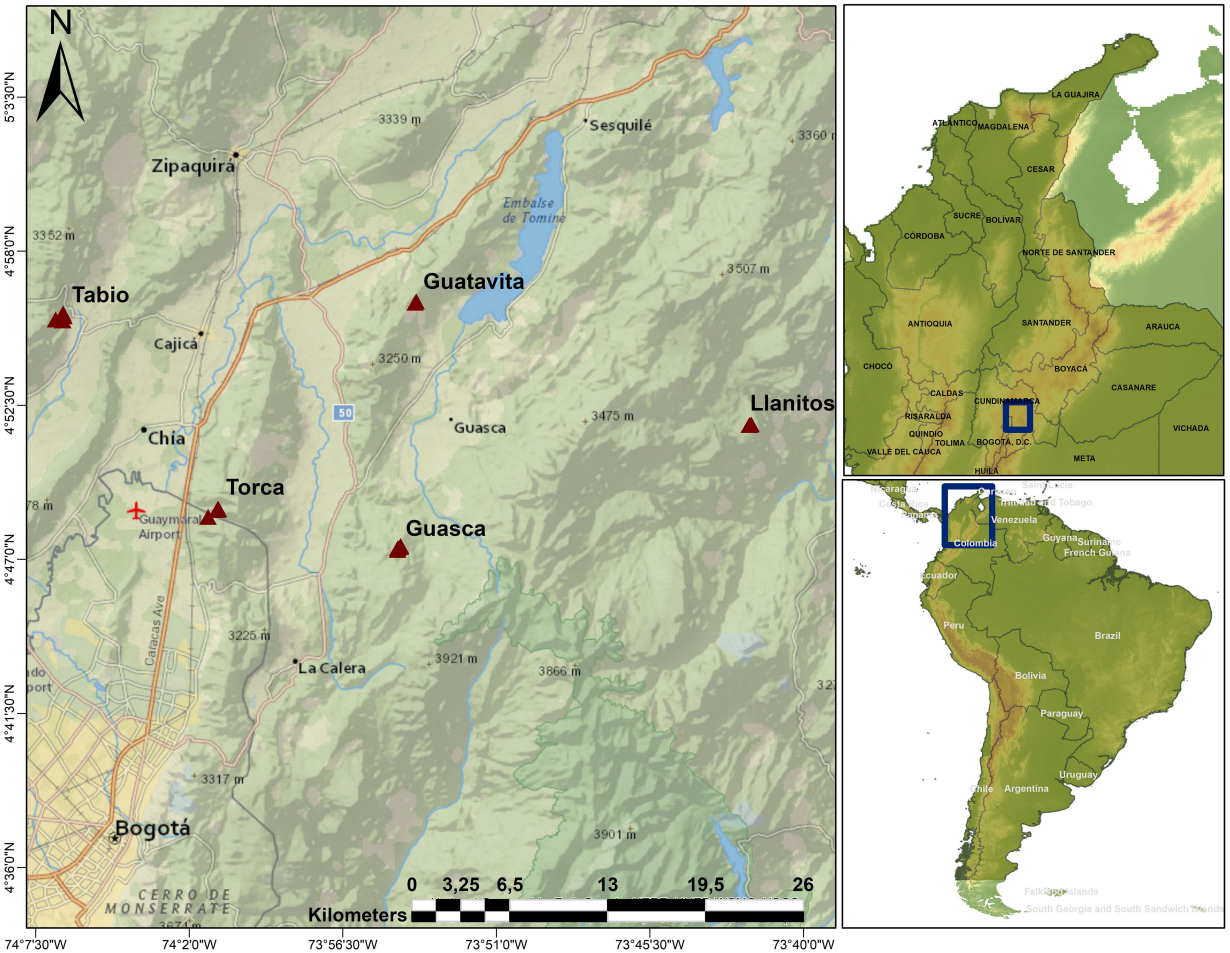
Study area. Triangles show the locations of the upper Andean tropical forests plots where sampling took place. Llanitos is a site within the municipality of Guatavita, where some species were sampled in 50x50 m plots.

This study was conducted in the Eastern Colombian Andes (**Figure 1**). The Andean region of Colombia covers 24.5% of the country (Etter and van Wyngaarden, 2000), it is the most populated region, with almost the 70% of the population, and harbors the economic core of the country (Departamento Administrativo Nacional de Estadística (DANE), 2018). As such, the Andes of Colombia have been heavily transformed by human activities. The study was carried out in the Cundiboyacense Highplain, which includes the Capital city of Bogotá and its surrounding municipalities. In these peri-urban sites, cattle raising, agriculture and mining are the dominant economic activities (Montañez et al., 1994; Antonio-Fragala and Obregón-Neira, 2011). Average annual atmospheric temperature in the area is 14 °C, and mean annual precipitation varies from 600 mm in the center of the mountain range to 1200 mm in the western part (Clerici et al., 2016). Precipitation typically displays a bimodal pattern with two rainy seasons: one occurring from April to June, and another between September and November (Mendoza and Etter, 2002).

We established 14 20x20 m plots and two 50x50 m plots in secondary and mature Andean Mountain forests at elevations ranging from 2685 to 3140 m. These plots are part of a larger network consisting of 36 20x20 m plots and eight 50x50 m plots. Forests were classified as secondary or mature by local experts, based on their structural attributes (basal area, tree height, tree density) and species composition. We mostly focused on the 14 20x20 m plots (see below), but we also sampled WD in some species in the 50x50m plots (Llanitos). The plots are on private properties/reserves, distributed in three municipalities (Tabio, Guasca and Guatavita) and one site (Torca) located close to the boundaries of the Capital District of Bogotá (**Figure 1**). In the 20x20 m plots, we tagged and identified all individuals with a diameter at ‘ankle’ height (DAH, 5 above the soil surface; Hurtado-M et al., 2021) larger or equal to 5 cm. We used this approach to include shrubs that usually branched near the ground or to include individuals with twisted stems. We also recorded diameter at breast height (DBH) to estimate biomass using allometric equations (see below). In the 50x50 m plots, we only sampled individuals with a DBH larger or equal to 10 cm. In the 20x20 m plots, the five dominant plant families were: Ericaceae, Melastomataceae, Cunoniaceae, Primulaceae, and Asteraceae, representing 57.7% of individuals. The five dominant species were: *Weinmannia tomentosa* Linnaeus filius 1782, *Cavendishia bracteata* Hoerold 1909, *Miconia squamulosa* Triana 1872, *Myrcianthes leucoxyla* McVaugh 1963, and *Myrsine latifolia* Spreng. 1824, with 46.16% of individuals. In total, 79 shrub and tree species were identified in the permanent plots. We also included seven common species found around the plots to complete a dataset of 86 plant species. Plant nomenclature was based on the Tropicos® Database from the Missouri Botanical Garden (https://www.tropicos.org/) and the World Flora Online Database (http://www.worldfloraonline.org).

### Forest structural attributes and successional gradient

In each of the 14 20x20 m plots (**Table 1**) we quantified the percentage of shrubs (% shrubs), stem basal area (m^2^) based on DAH, canopy height (m) and AGB (Mg C ha^-1^); we assumed that average C content was 0.5 (Petersson et al., 2012). Species were classified as shrubs if they had permanent wood, ramified from their basal part or below the soil surface, had no distinctive trunk, and their height was lower than 5 m (Orsham, 1989; Rundel, 1991; Mahecha et al., 2012). To quantify AGB we measured DBH for all individuals with a DAH higher or equal to 5 cm. AGB per plot was estimated as the average of two allometric equations that are specifically formulated for Andean montane forests by (1) Sierra et al. (2007) and (2) Pérez and Díaz (2010), respectively:

**Table 1:** Characteristics of the 14 permanent plots in UATF, in Colombia.

Location	Plot ID	Succession	Longitude	Latitude	Elevation (m)	Canopy openness (%)	LAI	AGB (Mg C ha^-1^)
Guatavita	1	Secondary	4.936032208	-73.8983992	3036	17.89	2.52	14.24
	2	Secondary	4.936838381	-73.8977293	3028	13.14	2.67	12.15
Guasca	3	Mature	4.788977177	-73.9088368	3140	6.86	3.84	85.83
	4	Secondary	4.791296415	-73.9071906	3086	6.21	4.38	40.27
	5	Mature	4.790034406	-73.9087034	3107	9.62	3.17	85.26
	6	Secondary	4.790724746	-73.9071956	3095	9.38	3.29	28.17
Tabio	7	Secondary	4.928016186	-74.1081094	2696	5.85	3.39	37.70
	8	Secondary	4.929730331	-74.108633	2708	10.90	2.88	24.93
	9	Mature	4.926100402	-74.113118	2821	4.01	3.97	66.71
	10	Mature	4.925467632	-74.1087721	2685	7.68	3.36	56.46
Torca	11	Mature	4.813520516	-74.0162575	2946	7.35	3.08	110.76
	12	Mature	4.813315707	-74.0158326	2966	11.42	2.71	73.38
	13	Secondary	4.808671143	-74.0219939	2709	7.73	3.19	41.19
	14	Mature	4.812753333	-74.0163478	2954	7.89	3.16	103.76

LAI, Leaf Area Index; AGB, Aboveground biomass (Mg C ha^-1^).


(1) $$AGB=0.107314\;\cdot \;DBH^{\;2.422}$$

(2) $$AGB=0.190024\;\cdot \;DBH^{\;2.20295}$$

Considering that AGB is a good indicator of forest recovery (Brown and Lugo, 1990; Garnier et al., 2004; Lohbeck et al., 2015a; Garnier et al., 2016), we used it as a proxy of ecological succession altogether with canopy height (h, m), and basal area calculated from DAH (m^2^). Canopy height was calculated as the weighted average of the upper quartile of all tree height measurements.

To measure net primary production (NPP; Mg C ha^-1^ y^-1^), we collected litterfall in 0.25 m^2^ litter traps in the 14 20x20 m plots (10 per plot, 140 in total). The trap frames were made of ½ inch PVC tubes and covered with a 1 mm nylon mesh screen; traps were elevated ca. 1 m above ground. Litter was collected between May 2016 and April 2017 every 15-20 days to minimize loss by decomposition. Litter samples were oven-dried at 60 °C until constant weight and then weighed with an analytical scale. Branches with a diameter above 1 cm were excluded, and branches with a diameter lower than 1 cm, but wider than the trap were cut to the length of the traps. We also measured stem growth by taking DBH measures between 2013 and 2016. NPP was calculated as the sum of annual litter production and stem biomass production; dead trees and new recruits were treated following the methodology of Clark et al. (2001).

Tree or shrub heights were measured with a clinometer or a telescopic measuring rod. Hemispherical photographs were taken using a Canon Inc. EW-77 digital camera with a 180° Fisheye Zoom Lens (EF 8-15mm 1:4, Japan). Nine hemispherical photographs were taken per plot and processed with Gap Light Analyzer 2.0 (Frazer et al., 1999) to obtain LAI and canopy openness. Plot LAI and canopy openness are reported as average values per plot (**Table 1**).

### WD measurements

In 2019, we measured WD for all species found in the permanent plots, plus seven woody species sampled in the surrounding areas (**Figure 1**) following standardized methods (Pérez-Harguindeguy et al., 2013). Wood samples were collected from stems using a 5-mm increment borer. Samples were sapwood taken at 1.3 m for both trees and shrubs. Some shrubs had no defined stem at 1.3 m and sampling took place below that height, but never lower than 1.1 m. An average of five individuals were sampled per species. Yet, for dominant species we collected up to 12 individuals, while for some rare species we sampled between one and three individuals. For each wood sample, we measured green volume with the water-displacement method, and then oven-dried it at 105 °C, during 72 h, to obtain wood dry mass (Chave et al., 2005). WD (g/cm^-3^) was calculated as the ratio between dry mass and green volume (Williamson and Wiemann, 2010; Oliveira et al., 2019).

### Data analyses

To characterize interspecific variation in WD for our study area, we plotted a kernel density function of our data, and compared it to global (Zanne et al., 2009) and regional density functions, including one in the Neotropics (Chave et al., 2006) and one in a lowland tropical forest of French Guiana from the BRIDGE project (Baraloto et al., 2010). We also explored variation in WD between families via one-way Kruskal-Wallis test. To compare WD between life forms (trees vs shrubs), we performed a nonparametric Mann Whitney test for pairwise groups. This test was also used for pairwise comparisons of WD between UATF and each of the three databases.

To examine whether forest attributes are related to WD at the community level, we calculated CWMwd for each of the 14 plots using species mean WD values weighted by species biomass following Garnier et al. (2004). To compare CWMwd to forest structural and productivity variables we used reduced major axis regression (RMA) that models residual variation of both axes (Pearson, 1901; Warton et al., 2012). We regressed CWMwd against AGB, basal area, canopy height, aboveground NPP, productivity of stems (i.e., annual biomass gain of stems), litterfall production and % shrub individuals in plots. All variables were log_10_ transformed before analyses. Statistical analyses were performed in Rwizard 4.3 (Guisande et al., 2014) and PAST 4.1 (Hammer et al., 2001).

## Results

### WD variation in UATF

**Figure 2:**
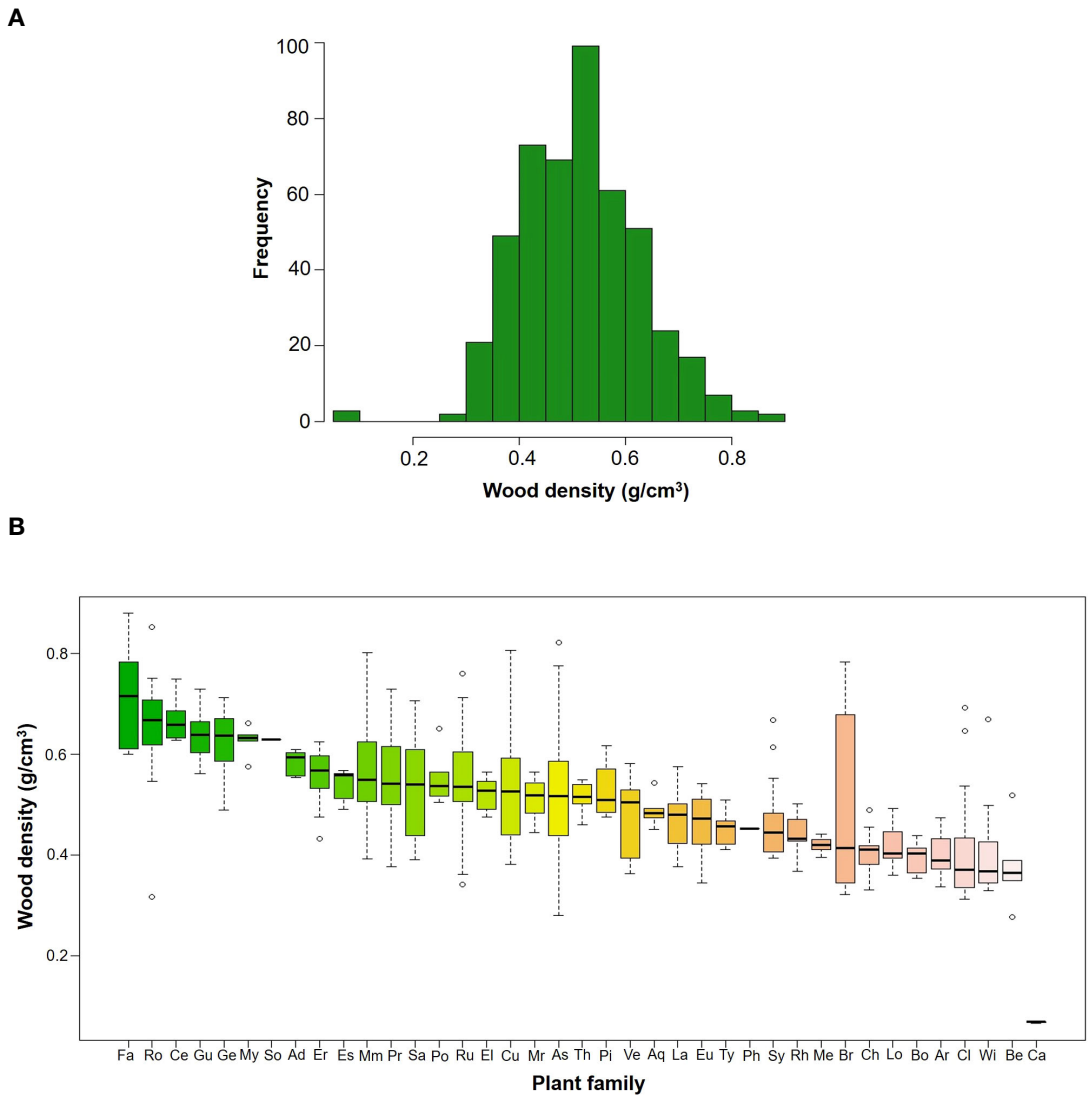
**(A)** Frequency histograms of WD by species in upper Andean tropical forests **(B)** Box plots of WD by families; the horizontal line is the median, the box is 50% of data, while the whisker extends to the furthest data point in each wing that is within 1.5 times the Interquartile range. Family acronyms are found in **Table 2**.

**Figure 3:**
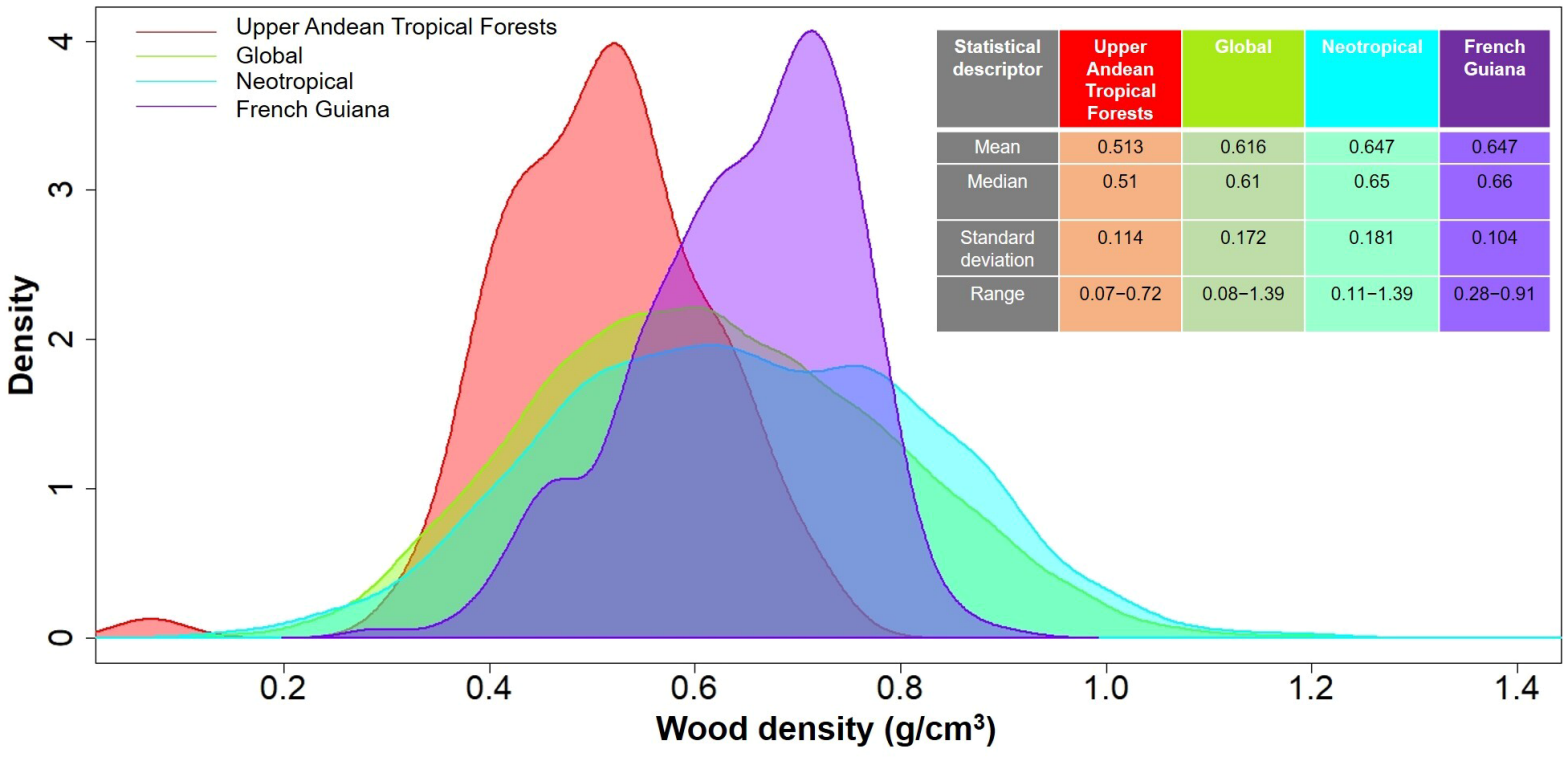
Density plots comparing wood density in upper Andean tropical forests, a lowland tropical rainforest in French Guiana (Baraloto et al., 2010), the Neotropics (Chave et al., 2006), and a global database (Zanne et al., 2009). Mean, median, standard deviation and range are shown on the upper right side.

In total, we measured WD in 481 individuals that belonged to 86 species, 59 genera and 39 families (**Table 2**). Species mean WD was 0.513 ± 0.114 g/cm^3^ and ranged from 0.068 (*Vasconcellea pubescens* with individuals as low as 0.065 g/cm^3^) to 0.718 (*Ulex europeus* with individuals up to 0.879 g/cm^3^) (**Figure 2A**). WD varied between families (H=220.75; df=38; p<0.0001), being Caricaceae, Betulaceae, Winteraceae, Clethraceae and Araliaceae the families with the lowest mean WD values (0.068-0.399 g/cm^3^), while Gentianaceae, Clusiaceae, Celastraceae, Rosaceae, and Fabaceae showed the highest values (0.619-0.718 g/cm^3^) (**Figure 2B**). The distribution of WD from UATF had a lower mean, a narrower range, and a smaller standard deviation than the global and Neotropical WD distributions (**Figure 3**). UATF also had a lower mean that a lowland tropical forest in French Guiana, but similar standard deviation and range. The overlap with the other datasets was: 66.59% (global), 59.18% (Neotropical) and 50.83% (lowland tropical forests from French Guiana). Significant differences were found in the pairwise comparisons between WD of UATF and the global (U= 222593; p<0.0001), Neotropical (U=55450; p<0.0001) and lowland tropical forests from French Guiana (U=6604; p<0.0001).

**Table 2:** List of species and their corresponding wood density in upper Andean tropical forests.

FAMILY/species	Acronym	N	Mean	SD	Range
**AQUIFOLIACEAE**	Aq	5	0.489	0.034	0.451-0.483
*Ilex kunthiana* ●		5	0.489	0.034	0.451-0.483
**ARALIACEAE**	Ar	11	0.399	0.046	0.336-0.472
*Oreopanax bogotensis* ●		6	0.396	0.040	0.385-0.459
*Oreopanax incisus* ●		5	0.403	0.057	0.336-0.472
**ASTERACEAE**	As	49	0.525	0.109	0.279-0.821
*Ageratina asclepiadea* ▲		5	0.511	0.072	0.391-0.586
*Ageratina fastigiata* ▲		5	0.568	0.077	0.441-0.639
*Ageratina glyptophlebia* ▲		6	0.682	0.084	0.563-0.775
*Baccharis macrantha* ▲		5	0.519	0.031	0.469-0.552
*Barnadesia spinosa* ▲		5	0.405	0.093	0.279-0.528
*Critoniopsis bogotana* ●		5	0.470	0.058	0.414-0.532
*Diplostephium rosmarinifolium*●		6	0.625	0.105	0.513-0.821
*Monticalia pulchella* ▲		5	0.491	0.033	0.458-0.531
*Verbesina arborea* ●		7	0.437	0.041	0.381-0.506
**BETULACEAE**	Be	6	0.377	0.079	0.276-0.517
*Alnus acuminata* ●		6	0.377	0.079	0.276-0.517
**BORAGINACEAE**	Bo	5	0.395	0.035	0.353-0.438
*Cordia cylindrostachya* ●		5	0.395	0.035	0.353-0.438
**BRUNELLIACEAE**	Br	7	0.509	0.197	0.321-0.782
*Brunellia acutangula *●		5	0.565	0.207	0.341-0.782
*Brunellia propinqua *●		2	0.367	0.065	0.321-0.413
**CARICACEAE**	Ca	3	0.068	0.002	0.065-0.069
*Vasconcellea pubescens *●		3	0.068	0.002	0.065-0.069
**CELASTRACEAE**	Ce	5	0.671	0.049	0.628-0.749
*Maytenus laxiflora *●		5	0.671	0.049	0.628-0.749
**CHLORANTHACEAE**	Ch	19	0.402	0.042	0.331-0.488
*Hedyosmum crenatum* ●		9	0.411	0.043	0.331-0.488
*Hedyosmum parvifolium* ●		5	0.415	0.039	0.355-0.454
*Hedyosmum* sp.●		5	0.372	0.032	0.331-0.411
**CLETHRACEAE**	Cl	15	0.413	0.120	0.311-0.692
*Clethra fimbriata* ●		6	0.331	0.024	0.311-0.371
*Clethra lanata* ●		6	0.388	0.036	0.354-0.437
*Clethra repanda* ●		3	0.626	0.080	0.537-0.692
**CLUSIACEAE**	Gu	12	0.637	0.049	0.561-0.729
*Clusia ducu* ●		3	0.613	0.045	0.561-0.644
*Clusia multiflora* ●		9	0.645	0.049	0.573-0.729
**CUNONIACEAE**	Cu	40	0.517	0.092	0.381-0.805
*Weinmannia auriculata* ●		10	0.543	0.130	0.381-0.805
*Weinmannia pinnata* ●		3	0.556	0.044	0.521-0.605
*Weinmannia tomentosa* ●		9	0.487	0.090	0.397-0.636
*Weinmannia rollotti* ●		8	0.515	0.063	0.404-0.626
*Weinmannia balbisiana* ●		10	0.509	0.080	0.398-0.599
**ELAEOCARPACEAE**	El	6	0.522	0.035	0.475-0.564
*Vallea stipularis* ●		6	0.522	0.035	0.475-0.564
**ERICACEAE**	Er	20	0.559	0.050	0.432-0.624
*Bejaria resinosa* ▲		5	0.587	0.013	0.568-0.600
*Cavendishia bracteata*▲		5	0.555	0.041	0.504-0.596
*Cavendishia nitida* ▲		5	0.552	0.036	0.506-0.604
*Macleania rupestris* ▲		5	0.542	0.086	0.432-0.624
**ESCALLONIACEAE**	Es	5	0.538	0.035	0.490-0.568
*Escallonia discolor* ●		5	0.538	0.035	0.490-0.568
**EUPHORBIACEAE**	Eu	10	0.458	0.066	0.343-0.541
*Croton bogotanus* ●		10	0.458	0.066	0.343-0.541
FABACEAE*	Fa	5	0.718	0.118	0.599-0.879
Ulex europaeus* ▲		5	0.718	0.118	0.599-0.879
**GENTIANACEAE**	Ge	5	0.619	0.086	0.489-0.712
*Macrocarpaea glabra* ▲		5	0.619	0.086	0.489-0.712
**LAURACEAE**	La	19	0.469	0.053	0.376-0.574
*Aiouea dubia* ●		6	0.468	0.043	0.393-0.508
*Nectandra* sp. ●		1	0.432	NA	NA
*Ocotea calophylla* ●		7	0.486	0.070	0.376-0.574
*Ocotea sericea* ●		2	0.441	0.055	0.401-0.479
*Persea* sp.●		3	0.466	0.046	0.414-0.504
**LORANTHACEAE**	Lo	5	0.419	0.051	0.360-0.492
*Gaiadendron punctatum*●		5	0.419	0.051	0.360-0.492
**MELASTOMATACEAE**	Mm	42	0.572	0.091	0.392-0.802
*Bucquetia glutinosa* ▲		6	0.531	0.052	0.480-0.599
*Miconia cundinamarcensis*●		7	0.554	0.065	0.436-0.640
*Miconia elaeoides* ●		5	0.507	0.069	0.392-0.562
*Miconia ligustrina* ▲		5	0.564	0.050	0.517-0.624
*Miconia squamulosa* ▲		7	0.723	0.050	0.664-0.802
*Tibouchina lepidota* ●		7	0.557	0.090	0.462-0.698
*Axinaea macrophylla* ●		5	0.530	0.039	0.489-0.584
**MELIACEAE**	Me	5	0.419	0.018	0.394-0.440
*Cedrela montana* ●		5	0.419	0.018	0.394-0.440
**MYRICACEAE**	Mr	12	0.514	0.040	0.444-0.565
*Morella parvifolia* ▲		5	0.513	0.031	0.468-0.548
*Morella pubescens* ▲		7	0.514	0.048	0.444-0.565
**MYRTACEAE**	My	5	0.626	0.032	0.574-0.660
*Myrcianthes leucoxyla*●		5	0.626	0.032	0.574-0.660
**PHYLLANTHACEAE**	Ph	1	0.453	NA	NA
*Hieronyma rufa* ●		1	0.453	NA	NA
**PIPERACEAE**	Pi	10	0.527	0.050	0.474-0.617
*Piper bogotense* ▲		10	0.527	0.050	0.474-0.617
**PODOCARPACEAE**	Po	5	0.554	0.058	0.503-0.650
*Podocarpus oleifolius*●		5	0.554	0.058	0.503-0.650
**PRIMULACEAE**	Pr	30	0.551	0.090	0.376-0.729
*Cybianthus* sp.▲		9	0.533	0.048	0.455-0.614
*Cybianthus iteoides* ▲		1	0.499	NA	NA
*Myrsine coriacea* ●		10	0.489	0.082	0.376-0.611
*Myrsine dependens* ●		5	0.635	0.092	0.535-0.729
*Myrsine latifolia* ●		5	0.633	0.044	0.575-0.696
**RHAMNACEAE**	Rh	10	0.439	0.037	0.367-0.501
*Frangula goudotiana* ●		5	0.438	0.024	0.407-0.471
*Frangula sphaerosperma* ●		5	0.440	0.051	0.367-0.501
**ROSACEAE**	Ro	16	0.654	0.115	0.317-0.852
*Hesperomeles goudotiana* ●		5	0.628	0.030	0.593-0.667
*Hesperomeles obtusifolia* ●		1	0.317	NA	NA
*Prunus buxifolia* ●		10	0.701	0.079	0.545-0.852
**RUBIACEAE**	Ru	22	0.543	0.113	0.340-0.760
*Palicourea angustifolia*▲		6	0.568	0.059	0.506-0.658
*Palicourea demissa* ▲		5	0.406	0.075	0.340-0.528
*Palicourea lineariflora* ▲		6	0.525	0.064	0.416-0.604
*Psychotria boqueronensis*▲		5	0.672	0.080	0.559-0.760
**SALICACEAE**	Sa	10	0.535	0.108	0.390-0.706
*Abatia parviflora* ▲		5	0.443	0.046	0.390-0.502
*Xylosma spiculifera* ●		5	0.628	0.051	0.576-0.706
**SOLANACEAE**	So	1	0.629	NA	NA
*Sessea corymbosa* ▲		1	0.629	NA	NA
**SYMPLOCACEAE**	Sy	17	0.465	0.079	0.393-0.668
*Symplocos mucronata* ●		12	0.455	0.068	0.402-0.613
*Symplocos theiformis* ●		5	0.486	0.108	0.393-0.668
**THEACEAE**	Th	5	0.513	0.035	0.459-0.548
*Gordonia* sp.●		5	0.513	0.035	0.459-0.548
**THYMELACEAE**	Ty	6	0.453	0.035	0.410-0.508
*Daphnopsis caracasana* ●		6	0.453	0.035	0.410-0.508
**VERBENACEAE**	Ve	16	0.478	0.079	0.363-0.580
*Citharexylum sulcatum* ●		5	0.543	0.046	0.488-0.580
*Duranta mutisii* ▲		5	0.522	0.007	0.510-0.529
*Lippia hirsuta* ●		6	0.386	0.023	0.363-0.421
**VIBURNACEAE**	Ad	5	0.583	0.026	0.553-0.609
*Viburnum triphyllum* ●		5	0.583	0.026	0.553-0.609
**WINTERACEAE**	Wi	11	0.406	0.102	0.328-0.668
*Drimys granadensis* ●		11	0.406	0.102	0.328-0.668

N = number of individuals; Mean = arithmetic mean; SD = standard deviation; Range = lowest and highest values. NA = not applicable for standard deviation or range because rare species had one individual in our sampling. For life form: ● = tree species and ▲ = shrub species. * Denotes an invasive species.

### Life form and WD variation

**Figure 4:**
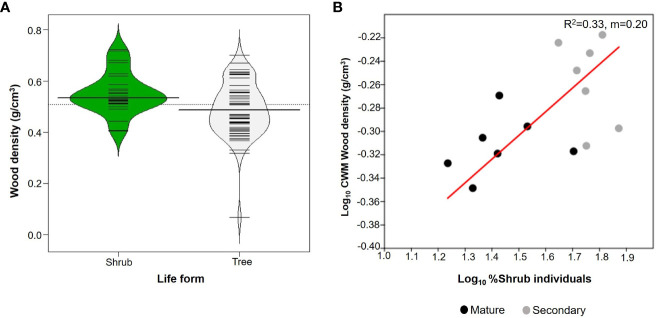
**(A)** Beanplots comparing shrubs (27 species) and trees (59 species) from upper Andean tropical forests plots. Each horizontal line represents the mean value of a species. **(B)** Reduced major axis regression between the % of individuals that are shrubs in each individual plot and community weighted mean (CWM) of wood density per plot. Values on the right corner of the plot are the coefficient of determination (R^2^) and slope (m). Grey circles correspond to secondary forests and black circles to mature forests plots.

Shrubs comprised 27 species whereas there were 59 species of trees (**Table 2**). Trees had an average WD of 0.488 ± 0.104 g/cm^3^ whilst for shrubs it was 0.552 ± 0.095 g/cm^3^. Shrubs had significantly higher mean values of WD than trees (W=1095; p=0.005; n=86; **Figure 4A**), although the two distributions overlapped considerably. In addition, the proportion of shrubs per plot was positively related to CWMwd (R^2 ^= 0.33; p=0.03; CI =[0.127, 0.273]; n=14; **Figure 4B**).

### WD, AGB and community attributes

AGB biomass ranged from 12.1 Mg C ha^-1^ to 110 Mg C ha^-1^, canopy openness between 5.85 to 17.89, and LAI between 2.52 and 4.38 (**Table 1**). On average, old-growth forests had an AGB of 83.16 Mg C ha^-1^ ( ± 18.87), a canopy openness of 7.83 (± 2.52), and a LAI of 3.32 ( ± 0.47), while secondary forests presented an AGB of 28.37 Mg C ha^-1^ ( ± 11.63), a canopy openness of 10.15 (± 4.53), and a LAI of 3.18 ( ± 0.67). AGB was significantly higher in mature forests than secondary ones (U= 28; p=0.017), but canopy openness (U=22; p= 0.17) and LAI (U=15; p=0.86) did not show any differences between successional stages.

**Figure 5:**
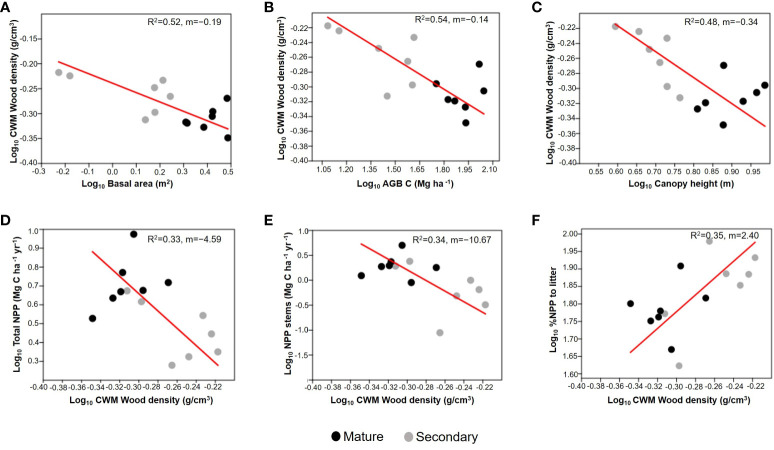
Reduced major axis regressions of community weighted mean (CWM) of wood density vs. basal area (m^2^; **A**), aboveground biomass (AGB; Mg C ha^-1^; **B**), and forest canopy height (average of the upper quartile of tree height; **C**); each point correspond to a plot. The bottom panels represent CWM of wood density vs. aboveground net primary productivity (Total NPP; Mg C ha^-1^ y^-1^; **D**), stem biomass growth (NPP stems; Mg C ha^-1^ yr^-1^; **E**), and the % of aboveground NPP attributed to leaf litter (% NPP to litter; **F**). Values on the right corner of each graph are the coefficient of determination (R^2^) and slope (m). All variables are log_10_ transformed. Grey circles correspond to secondary forests and black circles to mature forests plots.

We found significant negative RMA relationships between CWMwd and three variables related to forest successional status: basal area (R^2^=0.52; p=0.003; CI=[-0.232,-0.063]; n=14; **Figure 5A**), AGB (R^2^=0.54; p=0.002; CI=[-0.168,-0.075]; n=14; **Figure 5B**), and canopy height (R^2^=0.48; p=0.006; CI=[-0.449,-0.198]; n=14; **Figure 5C**). Regarding ecosystem productivity, we found an inverse relationship between CWMwd and aboveground NPP (R^2^=0.33; p=0.031; CI=[-6.508, -1.849]; n=14; **Figure 5F**) as well as CWMwd and stem growth (R^2^=0.34, p=0.029; CI=[-15.942,-2.770]; n=14; **Figure 5D**), but a positive one between CWMwd and %NPP attributed to litter (R^2^=0.35; p=0.024; CI=[0.973, 3.364]; n=14; **Figure 5E**).

## Discussion

### WD in upper Andean tropical forests

Wood traits have been under-sampled above 2500 m in the Andean region, and previous studies have reported WD values for only a few species in UATF (e.g., Chave et al., 2006; Vasquez-Valderrama and Solorza-Bejarano, 2018; López-Camacho et al., 2020; González-Melo, 2021). Here, we presented a comprehensive dataset of WD for 53 additional plant species for this ecoregion. We found that species mean WD for UATF (0.513 ± 0.114 g/cm^3^) was significantly lower than that reported in a global WD database (0.616 g/cm^3^), in Neotropical forests (0.647 g/cm^3^), as well as for lowland tropical wet forests in French Guyana (0.647 g/cm^3^; **Figure 3**). The mean value that we reported falls in the light-weight wood category according to Riesco et al. (2019). Other studies have found that WD decreases with elevation and decreasing temperature (Williamson, 1984; Chave et al., 2009), which agrees with our results (**Figure 3**; **Table 2**). Lower WD at high altitude can have several non-exclusive explanations. First, in tropical mountain forests of the Ecuadorian Andes, Moser et al. (2011) reported a noticeable reduction in NPP above 2000 m and suggested that wood production can be environmentally constrained at these higher altitudes. At high elevation, soil nitrogen availability and atmospheric temperatures are lower, which can lead to a reduction in leaf area, and consequently a decrease in carbon gain (Moser et al., 2011; Homeier and Leuschner, 2020). Similarly, Leuschner et al (2007; 2013). found a remarkable increase in the allocation of NPP in roots at high elevations, and a lower investment in tree trunk biomass and AGB, suggesting nutrient limitation for tree growth in cold environments. Yet, estimations of forest carbon budget along an altitude gradient in Perú showed that the fraction of NPP allocated to stems and roots do not change with elevation, despite lower gross and net primary production at higher than at lower elevation (Malhi et al., 2017).

We suggest that, in addition, a lower WD in UATF can be related to a more open canopy. The understory of lowland tropical rain forests acts as an important filter that favor species with conservative ecological strategies with high WD (e.g., Kitajima, 1994; Alvarez-Clare and Kitajima, 2007; Poorter et al., 2021b). In contrast, vertical stratification in UATF is simplified because of lower height of emergent trees, lower tree stem diameter, and lower leaf area (Myster, 2021), which may increase the light transmittance to the understory. This agrees with our results as LAI in UATF was lower (mean=3.26, **Table 1**) compared to lowland forests (LAI=5.1; e.g., Moser et al., 2007), suggesting that selection for shade tolerance (and high WD) could be less pronounced in UATF. Moreover, lower canopy height as well as lower diameter of tree stems at higher elevation (Myster, 2021) likely reduces the needs to invest in higher mechanical strength (and hence higher WD; Chave et al., 2009). Lastly, although WD exhibits an important intra-specific variation in response to environmental conditions (Patiño et al., 2009), some studies have shown that this trait is highly influenced by phylogeny (Chave et al., 2006; Chave et al., 2009). Thus, low WD in Andean forests can also be influenced by environmental filters favoring certain taxonomic groups with lower WD.

*Ulex europaeus* was the only exotic species in our plots and had the highest WD of all the 86 species (**Table 2**). Note, however, that excluding it from our analyses did not alter the overall results, due to its low biomass in the plot where it was present (data not shown). *U. europaeus* is considered an aggressive invasive species worldwide (Lowe et al., 2000), including the Highlands of Colombia that have seen an important expansion in its range since the 1950’s (Vargas et al., 2009). Its invasive success has been related to high reproductive and survival rates, and high resource acquisition capacity (Beltrán and Cataño, 2014). This species comes from the Mediterranean coast and its high WD is likely related to its shrub life-form (see below) and its evolutionary origin from an arid biome. Yet, it will be interesting to determine if its high WD also contributes to its invasive potential in UATF.

### Life forms

Shrubs showed higher WD than trees (**Figure 4**), as has been reported in previous studies (Jacobsen et al., 2007; Wheeler et al., 2007). This difference could be partially explained by habitat preferences and life-history variations between shrubs and trees. In general, shrubs are common in low-resource environments, such as dry ecosystems or shaded forest understories, characterized by light, water, and/or nutrient constraints (Petit and Hampe, 2006; Martínez-Cabrera et al., 2011). Moreover, trees and shrubs may differ in their wood structures because of differences in hydraulic demands. For example, since shrubs transport water to shorter distances compared to trees, allocation of stem cross-sectional area to vessels is expected to be lower in shrubs than trees. Shrubs may, therefore, allocate a higher percentage of stem volume to fibers (Martínez-Cabrera et al., 2009), via higher fiber wall to lumen ratios and/or higher fiber fractions (Ziemińska et al., 2015).

### Relationship to successional gradient and forest structural attributes

**Figure 6:**
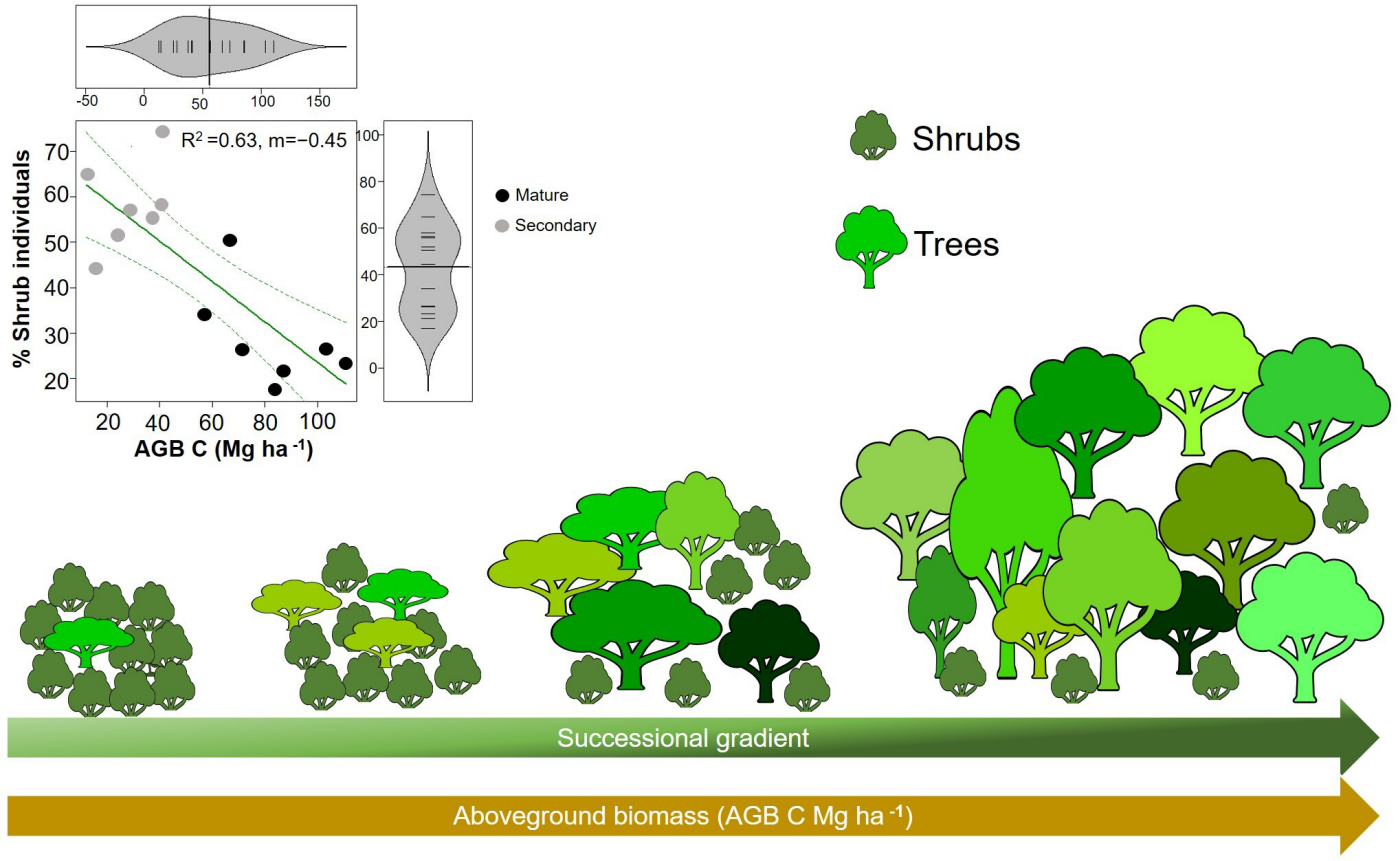
Illustration of changes in aboveground biomass and the percentage of shrubs along the successional gradient. Reduced major axis regression between the percentage of shrubs and aboveground biomass (AGB) in plots (p=0.0007; R^2^=0.63; n=14). Grey circles correspond to secondary forests and black circles to mature forests plots.

Our hypothesis that WD will increase during succession was not supported, and our results were in contrast with the pattern found on lowland tropical rain forests (Chazdon, 2014; Lohbeck et al., 2015b; Poorter et al., 2019; Poorter et al., 2021b). WD is expected to reflect a trade-off between growth and survival (Poorter et al., 2008; Chave et al., 2009). That is, species that invest in dense wood have higher survival in the understory due to an increase protection against herbivory and mechanical damage, while species that produce lighter wood tend to grow faster, via higher hydraulic conductivity and low stem construction costs, which allow them to take advantage of higher resource availability early in succession (King et al., 2006; Chave et al., 2009; Chua and Potts, 2018). Here, we found that early successional forests (i.e., with low AGB, basal area and height) had higher CWMwd than late successional forests (i.e., with high AGB, basal area and height). Interestingly, this result is similar to the pattern found in tropical dry forests where species with drought tolerance traits (i.e., high WD) are dominant in early succession as a response of water limitations and are gradually replaced by species with more acquisitive strategies during forest recovery (Lohbeck et al., 2015b; Poorter et al., 2019). In our study, higher CWMwd is largely related to a high abundance of shrubs (with higher WD than trees, **Figure 4B**) early in succession (**Figure 6**).

The high proportion of shrubs during the first stages of forest recovery can have several explanations. First, seed availability of tree species can be limited due to intense and prolonged land use in the region, which likely resulted in a disproportionate cutting of trees over shrubs. Second, shrubs tend to produce more seeds per square meter of canopy per year than trees (Moles et al., 2004; Moles et al., 2005), which favor their colonization in the landscape, especially in human-modified ecosystems. Thirdly, more harsh abiotic conditions may have filter out trees in the early stages of succession. A study conducted in the same plots showed that tree seedling composition was highly sensitive to edaphic conditions including pH, available phosphorous and calcium, which can be altered by anthropogenic pressures (Hurtado-M et al., 2021). Considering adverse conditions for seedlings in early-successional mountain forests, such as nutrient-poor soils (Vitousek et al., 1988; Tanner et al., 1998), harsh winds (Sugden, 1986), periodic drought stress (Van Steenis, 1972), and frost, it is likely that environmental filtering at these first stages can favor the establishment of shrubs with denser woods and higher stress tolerance, instead of trees with lighter woods. In line with this, some studies have found that shrubs tend to present not only higher WD (Larjavaara and Muller-Landau, 2012; Santiago et al., 2018), but also thicker roots than trees (Fortunel et al., 2012; Valverde-Barrantes et al., 2017), which can favor their establishment under less favorable abiotic conditions of drought and low fertility that can be present early in succession in human-disturbed UATF.

We also found that the percentage of aboveground NPP attributed to litter increased with CWMwd (**Figure 5F**). This result can again be attributed to the dominance of shrubs in early successional forests (**Figure 6**). Shrubs gained little biomass in their trunks because they attain their maximum height after a few years, so most NPP was related to litter production and less to stem growth (**Figures 5E, F**). Conversely, in old-growth forests the presence of shrubs was lower, and many trees in the plots had not attained their maximum height, resulting in proportionally more stem growth, which is reflected in both the increase of total NPP and the fraction of NPP attributed to stem biomass gain (**Figures 5D, E**). These results are important in the context of carbon cycling and forest succession as some studies have found that WD is inversely related to carbon gains (Hubau et al., 2020). Thus, old-growth forests from UATF with lower CWMwd can produce more biomass and act as significant carbon sinks, as has been suggested recently (Duque et al., 2021).

The large gap of information about functional traits of species from UATF call the attention for future research efforts. WD is particularly relevant in trait-based ecology given its link to species ecological strategies, community organization and biogeochemical carbon cycling (Evans et al., 2000; Chave et al., 2005; Van Gelder et al., 2006; Chave et al., 2009). The study of local or global factors that influence WD could help better predict the biomass stored in Andean forests. This is especially important under a global change scenario, where the impact of human-dominated landscapes and climate change in Andean ecosystems keep growing, but is still poorly understood. In the Colombian Andes, less than 25% of the original forest coverage is still present (Armenteras et al., 2003; Etter et al., 2021). Therefore, to ensure adequate biomass recovery of secondary forests, it is imperative to fill knowledge gaps on the major factors that can influence carbon storage such as WD.

## Conclusions

We measured WD in 86 species from UATF, which represent, for 53 of them, their first known report. These data could be incorporated into regional and global databases, thus contributing to help filling an important gap for these ecosystems (Báez et al., 2022). UATF had a lower average WD than the global, neotropical or lowland French Guyana forest. This low WD can be related to lower productivity, higher allocation to roots, lower tree stature, and/or a weaker “shade filter” due to higher light availability in the understory of UATF. We also found that disturbance, dispersal, and establishment filters appear to have favored shrubs with higher WD in early successional forests. In contrast, old-growth forests had a lower CWMwd due to a decrease in the abundance of shrubs. The decrease in aboveground NPP with WD can also be partially attributed to change in the abundance of shrubs, which showed reduced annual biomass gain in their trunks and a large fraction of NPP being related to litter production. Overall, we found that WD is a key functional trait related to plant community assembly and carbon cycling along successional gradients. Further research should determine how WD is related to other components of the carbon cycle and to different functional traits. That will deepen our understanding of the evolution of ecological strategies in UATF species and help us make better predictions about the benefit of the restoration of these forests on ecosystem services.

## Data availability statement

The original contributions presented in the study are included in the article/supplementary material. Further inquiries can be directed to the corresponding author.

## Author contributions

DC-F: Writing – original draft, Writing – review & editing, Conceptualization, Formal Analysis, Investigation, Methodology, Data curation. AG-M: Conceptualization, Data curation, Investigation, Methodology, Writing – original draft. JP: Conceptualization, Investigation, Methodology, Writing – original draft, Formal Analysis, Funding acquisition, Project administration, Resources, Supervision, Validation, Visualization, Writing – review & editing.
